# Short-Term Treatment with Empagliflozin Resulted in Dehydration and Cardiac Arrest in an Elderly Patient with Specific Complications: A Case Report and Literature Review

**DOI:** 10.3390/medicina58060815

**Published:** 2022-06-16

**Authors:** Sopak Supakul, Yurika Nishikawa, Masanori Teramura, Tetsuro Takase

**Affiliations:** 1 Graduate School of Medicine, Keio University, Tokyo 160-8582, Japan; sopaksupakul@gmail.com; 2 Omori Red Cross Hospital, Tokyo 143-8527, Japan; yurika.nishikawa923@gmail.com; 3 Department of Cardiovascular Medicine, Ichinomiya Nishi Hospital, Aichi 494-0001, Japan; teramu1979@yahoo.co.jp

**Keywords:** sodium-glucose cotransporter-2 (SGLT-2) inhibitor, empagliflozin, dehydration

## Abstract

Empagliflozin is a sodium-glucose cotransporter-2 inhibitor widely used in the treatment of diabetes mellitus and heart failure. Our case study involved a 68-year-old patient who was admitted to the hospital because of a cerebral infarction. The patient was simultaneously diagnosed with diabetes mellitus and heart failure, for which empagliflozin was initiated. However, food and fluid intake were reduced due to poor appetite. In addition to the side effects of empagliflozin, the patient developed severe dehydration and cardiac arrest. Careful assessment of dehydration and preventive water intake is recommended in elderly patients and those with neurological deficits, especially when receiving empagliflozin.

## 1. Introduction

Empagliflozin (Jardiance^®^), a sodium-glucose cotransporter-2 (SGLT-2) inhibitor, is a novel medication for the treatment of type 2 diabetes mellitus (T2DM). It blocks the reabsorption of glucose from the proximal part of the nephron and effectively controls serum glucose levels. Thus, it is beneficial to treat diuresis by reducing excess water from the body [1]. Recent studies have suggested its effectiveness in patients with heart failure with preserved ejection fraction (HFpEF) [2]. The drug was also recently approved by the U.S. Food and Drug Administration (FDA) and recommended in the 2021 European Society of Cardiology (ESC) guidelines for use in heart failure with reduced ejection fraction (HFrEF) [3,4]. In addition, trials have shown that SGLT-2 inhibitors are effective in reducing the risk of myocardial infarction (MI), stroke, and primary outcomes of chronic kidney disease (CKD) [5,6].

Since many studies have suggested that SGLT-2 inhibitors, including empagliflozin, are beneficial for both diabetes mellitus and other cardiovascular diseases, they have now become widely used worldwide. Although many recent clinical trials suggested the safety of empagliflozin, the possible adverse effects of this medication still need to be carefully monitored, especially in patients with complications leading to low diet and fluid intake. Herein, we report the case of a 68-year-old man who was admitted to the hospital with multiple acute cerebral infarctions complicated with untreated diabetes mellitus (DM) and HFrEF. The patient was administered empagliflozin 10 mg/day for 31 days; however, he eventually suffered dehydration and cardiac arrest.

## 2. Case Presentation

**Figure 1:**
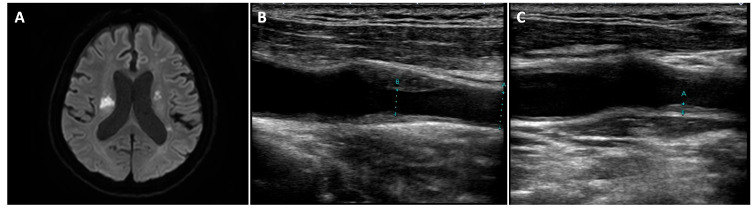
Diffusion-Weighted Magnetic Resonance Imaging (DW-MRI) of the brain and echocardiogram of the left and right carotid arteries. (**A**) Multiple infarcts in the left middle cerebral artery territory, right corona radiata, and right internal capsule shown in DW-MRI; (**B**,**C**) stenosis and plaque showed in the right (**B**) and left (**C**) carotid arteries.

A 68-year-old man was admitted to the hospital with left-sided paralysis and aphasia. The neurological assessment revealed reduced manual muscle testing scores on the left side of the body (upper limbs (5/0) and lower limbs (5/1)). Magnetic resonance imaging (MRI) revealed multiple infarcts in the left middle cerebral artery, right corona radiata, and right internal capsule. Further evaluation of carotid arteries by ultrasound showed 76% stenosis of the right carotid artery and moderate plaque on the left side (Figure 1). Although the patient presented with bilateral infarcts in the anterior circulation, no evidence of atrial fibrillation and patent foramen ovale was detected on a continuous electrocardiogram (ECG) monitoring and transesophageal echocardiography, respectively. Therefore, artery-to-artery embolism originating from the carotid arteries was considered the main cause of these infarctions.

The initial blood samples at the time of hospitalization showed normal complete blood count (CBC), normokalemia (K 3.8 mEq/L), normal kidney function (blood urea nitrogen (BUN) 15.3 mg/dL, creatinine 0.81 mg/dL, eGFR 72.8 mL/min/1.73 m^2^); however, hyperlipidemia (TG, 272 mg/dL), hyperglycemia (S-GLU 319 mg/dL, HbA1C 10.0%), and a moderately elevated level of BNP (97.2 pg/mL) were observed. Echocardiography revealed an ejection fraction of 39% with global hypokinesia. The patient had not been diagnosed with or received any treatment for DM and HF before the hospitalization.

After hospitalization, the patient started dual antiplatelet therapy with aspirin 100 mg/day, clopidogrel 75 mg/day, and statin 20 mg/day. Carvedilol 2.5 mg/day was administered to treat hypertension. In addition, linagliptin (dipeptidyl peptidase-4 (DPP-4) inhibitor) 5 mg/day and empagliflozin 10 mg/day were initiated to treat hyperglycemia and HF. The patient did not receive any other diuretics or medications with a diuretic effect. During hospitalization, the patient experienced angina pectoris. Coronary angiography was performed on day 18, revealing 90% stenosis of the left anterior descending artery. A drug-eluting stent was deployed on day 31 to treat the lesion. The patient was started on oral food and fluid intake on day 9 of hospitalization. However, the amount of consumption varied from 0 to 100% of the hospital meals depending on the appetite, and 500 mL to 1500 mL of intravenous (IV) fluid per day was continued to supplement his low oral intake. On day 33, his food intake of the meals improved to more than 50%; therefore, the IV fluid supplementation was discontinued.

**Figure 2:**
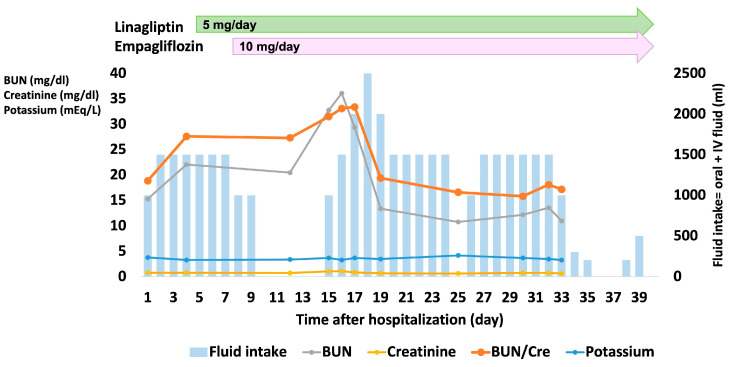
Clinical course during hospitalization. BUN—blood urea nitrogen; BUN/Cre—blood urea nitrogen/creatinine, IV: intravenous.

The patient’s water intake and laboratory findings related to dehydration and renal function, including BUN, serum creatinine, and potassium levels, are illustrated in Figure 2. Serum potassium was always within the normal range, while BUN/creatinine and creatinine levels increased on days 15 to 17, a few days after the IV fluid decreased. The number of times to urinate was constantly 2–5 times per day over the course of hospitalization, though accurate urine output was not measured, and the amount of urine per urination may have decreased. In addition, serum sodium levels were elevated to 149 mEq/L on days 15 to 17, but otherwise, they remained within the normal range (data not shown). It should be noted that blood tests were not properly performed during days 34–40 due to the national holiday period.

On day 40, the patient experienced cardiac arrest. The ECG showed asystole, and the patient did not respond to cardiopulmonary resuscitation (CPR). A blood sample taken while performing CPR showed significantly elevated liver enzyme (aspartate aminotransferase (AST) (>2000 U/L); alanine aminotransferase (ALT) (>2000 U/L)), lactate dehydrogenase (LDH) (>4000 U/L), creatinine kinase (>900 U/L), serum potassium (>10.0 mEq/L), phosphate (>20.0 mEq/L), BUN 122.6 mg/dL, and creatinine 9.88 mg/dL, which resulted from the end-organ damages due to organ hypoperfusion. Postmortem examination showed no evidence of stent thrombosis, coronary artery thrombosis, aortic rupture, massive pulmonary embolism, or any other abnormalities in major organs, such as the aorta, heart, and lungs, which could cause cardiac arrest. The cause of death was suspected to be associated with dehydration due to low food and fluid intake accompanied by empagliflozin treatment, which possibly led to acute kidney injury, hyperkalemia, and subsequent cardiac arrest.

## 3. Discussion

Given the therapeutic effects of empagliflozin on HF in patients with DM (Table 1), it has been widely used in many clinical settings worldwide. Empagliflozin is effective in reducing the risk of hospitalization for HF and cardiovascular death in diabetic patients with established cardiovascular disease [7] and HFpEF [2]. It also helps reduce the left ventricular end-diastolic volume index by 8.2 mL/m^2^ (95% confidence interval (CI) −13.7, −2.6) (*p* = 0.0042) and reduce the N-terminal pro-B-type natriuretic peptide by 28% (95% CI 2%, 47%) (*p* = 0.038) after 36-week treatment [8]. Thus, the mechanism of alleviating HF pathology could underlie the lower hospitalization rates. The medication also showed the same efficacy and adverse event profile in the Asian population compared to the non-Asian population [9]. Empagliflozin is currently recommended as pharmacological therapy for HFrEF patients by the FDA, 2021 ESC guidelines, and 2021 guidelines of the Japanese Circulation Society and Japanese Heart Failure Society (JCS/JHFS) [3,4,10].

For the treatment of Type 2 Diabetes Mellitus (T2DM), empagliflozin has a compelling effect in improving pancreatic β-cell functions and increasing insulin sensitivity [14]. Although using empagliflozin with insulin poses a risk of hypoglycemia, the relatively independent effects of the drug on pancreatic β-cell functions or insulin mechanistic pathways indicate the use of drugs in any stage of T2DM [15]. The drug also inhibits SGLT-2 more than 2500-fold compared to SGLT-1 [16]. This suggested a high affinity of the drug. In clinical trials, empagliflozin showed a dose-dependent effect in reducing glycemic levels; changes in baseline of HbA1C as −0.74% (95% CI −0.88, −0.59; *p* < 0.0001) for 10 mg dose and −0.85% (95% CI −0.99, −0.71; *p* < 0.0001) for 25 mg dose for a 24-week monotherapy [17]. Empagliflozin is also effective when administered as an add-on treatment to metformin and sulfonylurea [18], metformin [19], and pioglitazone [20]. Furthermore, similar to our case, a fixed-dose combination of empagliflozin 10 mg and linagliptin 5 mg showed a greater reduction in HbA1c baseline in diabetic patients with no significant adverse effects [21,22].

Regarding the complications of empagliflozin, no reported changes were observed in serum potassium or serum creatinine levels after short-term (7 days) treatment, although the eGFR slightly decreased after treatment [11]. In contrast, serum uric acid levels reduced after both short-term and long-term treatment [8,12]. In addition, the results of the EMPEROR-Preserved clinical trial indicated a greater risk of uncomplicated genital and urinary tract infections and hypotension in the empagliflozin treatment group [2]. Previous studies have also suggested a decrease in body weight and systolic blood pressure with the administration of empagliflozin [14,15,23]. Complications of other SGLT-2 inhibitors include serum electrolyte alterations that are not observed with empagliflozin, such as hyperkalemia and hypermagnesemia with canagliflozin [24]. In addition, changes in bone mineral density and bone fractures were observed in patients with moderate renal impairment receiving dapagliflozin [25]. Similar to our case report, some other studies also reported dehydration found in patients receiving empagliflozin [26,27]. Therefore, dehydration, especially in elderly patients or patients with morbidities receiving empagliflozin, should be carefully monitored. 

It is often difficult to detect dehydration and hypovolemia during physical examination, especially in elderly patients or those with neurological deficits. In these patients, common physical and functional changes such as anorexia, cognitive impairment, decreased feeling of thirst, and dysphagia are common [28], and the indicative physical findings for dehydration, such as muscle weakness, lethargy, and reduced activity levels, are often overlooked. Therefore, difficult-to-detect dehydration in a specific group of patients should be suspected by combining both physical and laboratory findings. Previously, Gross et al. (1992) identified a set of signs and symptoms that were best correlated with dehydration severity, such as tongue dryness, dryness of the mouth, upper body muscle weakness, and level of sunken eyes [29]. For the laboratory markers, creatinine and BUN/creatinine were shown to be useful indicators for dehydration [30]. Nevertheless, with all the suggested signs and laboratory findings, dehydration can still be difficult to detect. Therefore, preventive water intake in elderly patients is recommended. Chidester et al. (1997) established the standard adequate fluid intake criteria for elderly patients as 1500 mL for the first 20 kg and 15 mL for the remaining kg [31]. The European Society for Clinical Nutrition and Metabolism (ESPEN) guidelines also suggest at least 1.6 L and 2.0 L of drinks per day for elderly women and men, respectively [32]. The continuous monitoring of dehydration and preventive water intake in elderly patients who receive empagliflozin, or other SGLT-2 inhibitors, might prevent the serious side effects shown in our case. 

Finally, other possible causes of acute deterioration of this patient need to be discussed. The patient had been treated for coronary artery stenosis during hospitalization, and therefore the possibility of cardiac arrest due to acute coronary syndrome (ACS) was also possible. However, at the postmortem examination, the coronary arteries were thoroughly examined, and there was no evidence of coronary thrombosis. Additionally, diabetic ketoacidosis (DKA) was reported to associate with the use of SGLT2 inhibitors. The U.S. FDA and the European Medicines Agency (EMA) also warned about possible atypical DKA with SGLT2 inhibitors, as in several reported cases, patients’ blood sugar levels were only slightly increased compared to typical cases of DKA [33]. They recommend evaluation for the presence of acidosis when the patient shows any signs of ketoacidosis including difficulty breathing, nausea, vomiting, abdominal pain, confusion, and unusual fatigue or sleepiness. In our case, fasting and casual blood sugar levels before each meal were recorded throughout the hospitalization, and they were consistently normal or mildly elevated. However, due to the aphasia and declines in ADLs with paralysis in this patient, the indicative signs of DKA might have been difficult to detect, and the presence of ketoacidosis was not evaluated within a week before the deterioration. Thus, there is a possibility that DKA was presented in this patient, which is consistent with the presence of severe dehydration.

## 4. Conclusions

Collectively, our case report presents an elderly patient with neurological deficits that led to decreased food and fluid intake. Continuous treatment with SGLT-2 inhibitors may have contributed to dehydration and subsequent cardiac arrest. Although there are many advantages to using this treatment, it is important to note that there are individual risks that potentially lead to serious adverse effects, as shown in this case. Careful monitoring of elderly patients with neurological deficits who receive this medication is strongly recommended.

## Figures and Tables

**Table 1:** Summary of selected clinical trials showing effects of empagliflozin on heart failure in patients with diabetes mellitus.

No.	Targets	Number of Participants	Dose	Duration	Main Outcomes	Reference
1	Established CVD with T2DM	7200	10 or 25 mg/day	192 weeks	The risks of cardiovascular death, all-cause mortality, hospitalization for heart failure, and all-cause hospitalization were reduced in patients with and without prevalent kidney disease after Empagliflozin treatment. No significant hyperkalemia event was observed in the treatment group. Serum uric acid was lower in the treatment group.	[7]
2	Decompensated HF with T2DM	23	10 mg/day	7 days	Increased urine volume was observed on day 1 after the start of the treatment and returned to baseline on day 7. While there were no changes in serum potassium and creatinine on day 7, plasma neurohormone, including aldosterone and noradrenaline levels, increased. eGFR slightly decreased on day 7.	[11]
3	Chronic stable HF with T2DM	20	10 mg/day	14 days	The enhanced natriuretic effect of empagliflozin persists at day 14 without electrolyte alterations, neurohormone activation, and kidney dysfunction. Thus, Empagliflozin favors volume management in patients with HF and DM.	[12]
4	Chronic HFrEF with/without DM	1863	10 mg/day	27 months	The risk of cardiorenal outcomes was reduced after treatment with empagliflozin in addition to other HF therapy in HFrEF patients both with and without DM. There was no significant hyperkalemia, volume depletion, or acute renal failure among the treatment/placebo and DM/non-DM groups.	[13]
5	HFrEF with DM	105	10 mg/day	36 weeks	Empagliflozin is effective in reducing the left ventricular end-systolic and end-diastolic indexed volumes (6.0 and 8.2 mL/m^2^). The drug also reduced N-terminal pro-B-type natriuretic peptide by 28% after 36-week treatment.	[8]
6	HFpEF with/without DM	5988	10 mg/day	36 months	The number of deaths from cardiovascular causes or hospitalization due to HF was reduced in the empagliflozin group.	[2]
7	Asian participants with T2D and established ASCVD	1517	10 or 25 mg/day	3.1 years (Median follow-up)	Empagliflozin reduced the relative risk of the primary outcome of 3-point major adverse CV events (composite of CV death, non-fatal myocardial infarction, and non-fatal stroke), hospitalization for HF, and CV mortality. The medication efficacy and adverse event profile were similar between the Asian and Non-Asian populations.	[9]

ASCVD—atherosclerotic cardiovascular disease; CVD—cardiovascular disease; DM—diabetes mellitus; eGFR—estimated glomerular filtration rate; HF—heart failure; HFpEF—heart failure with preserved ejection fraction; HFrEF—heart failure with reduced ejection fraction; T2DM—type 2 diabetes mellitus.

## Data Availability

All data shown in this study are included in this published article.
